# Cancer Arising in a Duodenal Duplication Cyst: A Rare Genetic Anomaly

**DOI:** 10.7759/cureus.14782

**Published:** 2021-04-30

**Authors:** Sarbjot Grewal, Neil Behniwal, Jasleen Kaur, Rupinder Mann, Ravi Rao

**Affiliations:** 1 Internal Medicine, Saint Agnes Medical Center, Fresno, USA; 2 Hospital Medicine, Saint Agnes Medical Center, Fresno, USA; 3 Hematology and Oncology, Saint Agnes Medical Center, Fresno, USA

**Keywords:** duodenal duplication cyst, malignancy

## Abstract

We report the finding of a rare diagnosis of a duodenal duplication cyst (DDC) resulting in malignancy. Duplication cysts are rare entities in itself but less than 5% arise from duodenum. Our case represents a rare case, but high suspicion and early resection may have prevented associated complications.

## Introduction

Duplication cysts are rare congenital malformation of the gastrointestinal tract with an estimated occurrence of one in 100,000 live births. The exact etiology behind the occurrence of these cysts is unknown. Several theories have been proposed to explain their existence [1]. The most common location of these cysts is in the distal small intestine. Duplication cysts that arise in the duodenum are uncommon and account for only 5-7% of all such cysts [1]. When duplication cysts arising from the duodenum are found, they are often found in childhood with local pressure symptoms (pain, nausea, vomiting, obstruction). Occasionally, these cysts are asymptomatic, and can be found incidentally in adulthood. Duplication cysts can rarely turn malignant, and a few such cases have been reported. Herein, we present the case of a patient who had a malignancy found to arise in a duodenal duplication cyst. The other rarity in our patient was that she was found to have an activating mutation in the BRIP (BRCA1 Interacting Protein 1) gene [2]. When she had progressive disease, the use of a targeted agent (PARP-inhibitor) resulted in a sustained response.

## Case presentation

**Figure 1:**
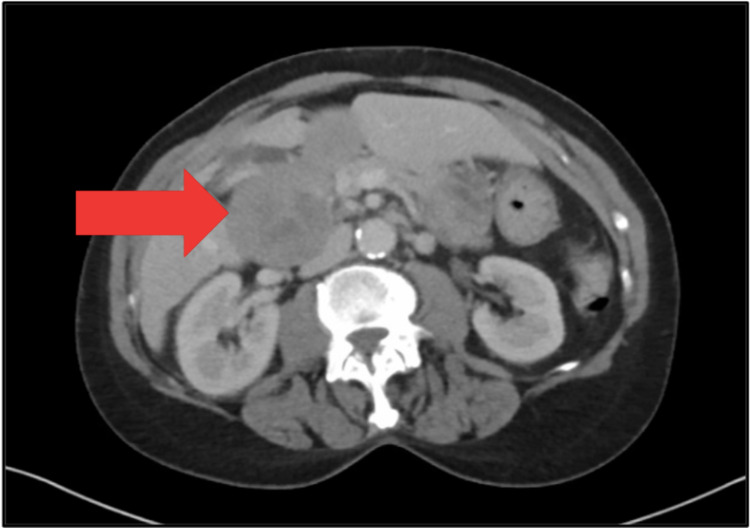
Axial cut of CT abdomen and pelvis showing duodenal cyst.

**Figure 2:**
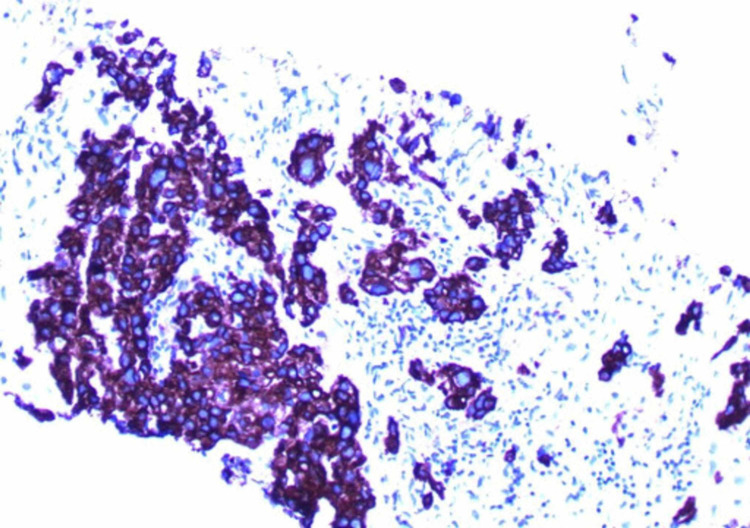
Tumor markers testing was positive for pancreatin.

**Figure 3:**
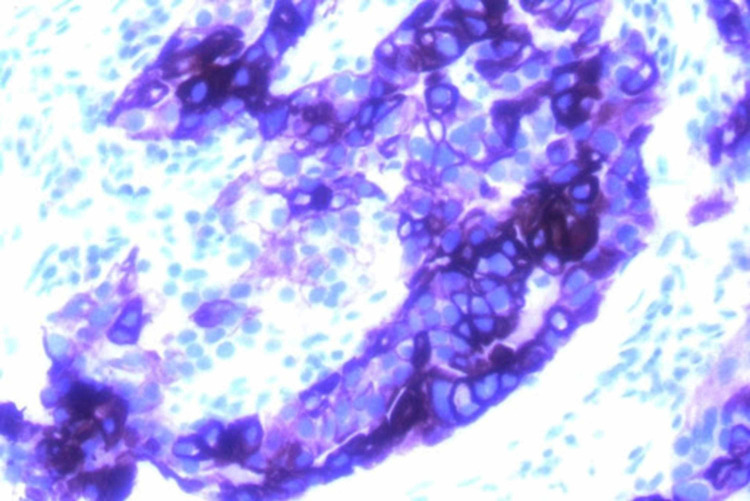
Tumor markers testing was positive for CK7. CK7: Cytokeratin 7

**Figure 4:**
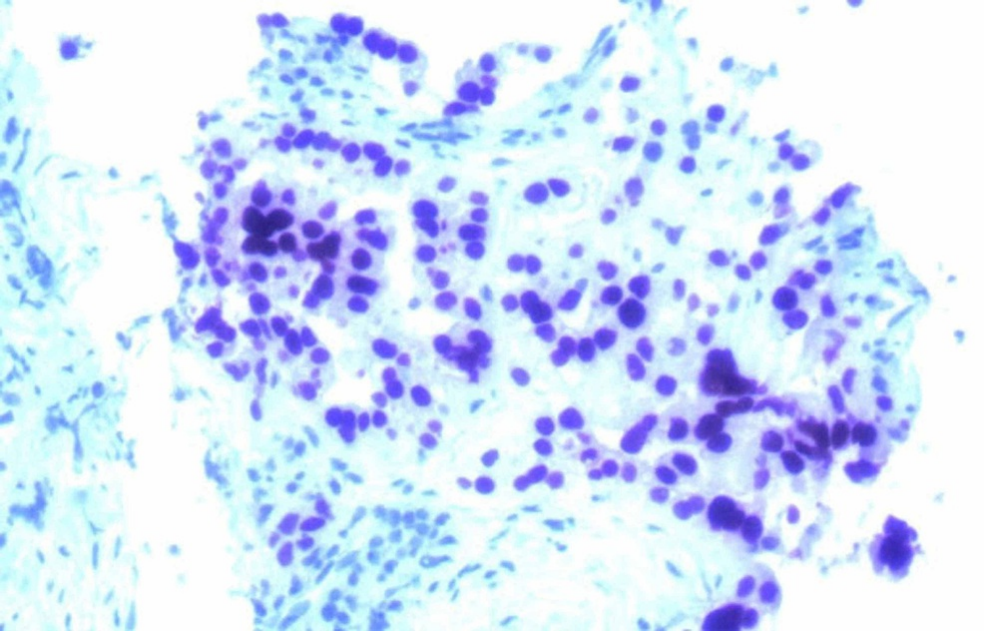
CK19 as well as focal positivity for CK20, and TTF-1. CK19: Cytokeratin 19; CK20: Cytokeratin 20; TTF-1: Thyroid transcription factor 1

A 69-year-old Caucasian woman presented to the ER with fevers and abdominal pain. A CT scan was performed, and identified diverticulitis with a small perforation (Figure 1). Incidentally, she was found to have enlarged masses in area of the porta hepatis and duodenum. She was treated conservatively for her infection with antibiotics and she recovered. Her family history was unremarkable, and she had been previously healthy. She was a non-smoker. Tumor markers were ordered, and while carcinoembryonic antigen (CEA), cancer antigen 19.9 (CA-19.9), cancer antigen 27.29 (CA 27.29) and cancer antigen 125 (CA-125) were normal, alpha-fetoprotein (AFP) was elevated. She then had a CT-guided percutaneous biopsy of the lesion. Pathology showed a poorly differentiated adenocarcinoma. The tumors tested were positive for pancreatin (Figure 2), cytokeratin 7 (CK7) (Figure 3), cytokeratin 19 (CK19) as well as focal positivity for cytokeratin 20 (CK20), and thyroid transcription factor 1 (TTF-1) (Figure 4) tumor markers and unequivocal for napsin A marker. This pattern was not completely definitive for any specific site, and a pathology second opinion was obtained. Patchy positivity for HepPar-1 was found suggesting hepatocellular carcinoma (HCC), but the confirmatory stain for Arginase-1 was negative.

Additional imaging including a positron emission tomography (PET) scan, MRI of the abdomen and testing by endoscopic ultrasound (EUS) did not reveal any site of primary in the liver, pancreas. EUS was performed and found the tumor to be adjacent to the duodenum, but again, no primary was noted on this study. A repeat biopsy of lymph node was performed and additional testing was done, including tissue analysis from the CancerTypeID© test (Biotheranostics, San Diego, CA). This test suggested a high probability that this was a pancreaticobiliary tumor. Given the unusual nature of presentation and absence of a primary, it was decided to attempt surgical exploration. She then underwent an exploratory laparotomy at which point, it appears that the tumor had arisen in a previously unsuspected duodenal duplication cyst. She underwent resection of the porta hepatic lymph node, and Whipple's procedure with pylorus preservation. Multiple (16/21) lymph nodes were involved. Post-operatively alpha-fetoprotein (AFP) levels decreased down to 45, but did not normalize. Given concerns that she may have residual cancer, she was offered postoperative chemotherapy. She received therapy with FOLFOX, and had multiple toxicities so therapy was stopped after three cycles. With this therapy, her AFP finally decreased down to the normal range. This suggested that she perhaps indeed did have residual disease post operatively, and had benefited from cytro-duction from surgery. She was therefore treated with a combination of radiation and infusional 5-FU with curative intent to the tumor field. Within two months after completion of radiation, she developed two liver metastases with a corresponding increase in her AFP. No biopsy was done, as these lesions were very definitively noted to be malignant on CT scans and also were noted to be PET avid. Next Generation Sequencing of the surgical sample revealed that she had an inactivating mutation in the BRIP gene (exon 10 rearrangement). There is data that mutations of this gene may lead to sensitivity to PARP inhibitors, so the patient was initiated on Olaparib therapy. She had a complete response to therapy, which has been maintained for more than 14 months and is ongoing.

## Discussion

The majority of duodenal duplication cysts (DDCs) are diagnosed in infancy or early childhood. Rarely they may remain asymptomatic and undiagnosed until adulthood and, depending upon their location, size and type, may present as bowel obstruction, bile duct obstruction, pancreatitis, bleeding or intussusception. Although DDCs are generally benign, malignant transformations inside them have already been reported [2-4]. The first case of its kind was reported after exploratory laparotomy in Japan in a 41-year-old female after she presented with hematemesis and pain in right hypochondrium [2]. Later in 1991, an adenocarcinoma and in 2006 carcinoid tumor in duplication cyst of the duodenum was reported in a 34-year-old woman. Characteristics of a duplication cyst include a smooth-muscle coat, an alimentary epithelial lining and an intimate attachment to the gastrointestinal tract. They occur at various levels of intestine, with great variations in shapes and sizes. In the intestine, jejunal duplications are most common, followed by gastric, duodenal duplication cysts being the rarest, accounting for 5% of the cases [5].

DDCs may present as a vague, often misleading clinical picture, thereby posing a diagnostic challenge for the clinician. Multiple case reports have reported an array of symptoms, including abdominal pain, nausea, anorexia, cachexia along with a clinical picture of recurrent acute pancreatitis or gastric outlet obstruction, or gastrointestinal bleeding, hemorrhagic ascites. Larger sized cysts result in compressive symptoms including malnutrition and weight loss, and may also result in compression of the pancreatic duct causing recurrent pancreatitis episodes with an unidentifiable cause [6-7].

The best diagnostic tools available are endoscopic ultrasound (EUS) which helps differentiate the duplication cyst by the “gut signature” or “the double-layer wall” sign of its wall. Computed tomography of the abdomen and pelvis may help delineating the lesion and size as well. For further differentiation, a technetium scan may be helpful as well in detecting the heterotopic gastric mucosa in cases that present with bleeding [6]. Other diagnostic modalities include magnetic resonance imaging (MRI), and gastroduodenoscopy, magnetic resonance cholangiopancreatography (MRCP). Endoscopic retrograde cholangiopancreatography (ERCP) is also recommended to visualize the pancreaticobiliary tract and determine its relationship with the cyst [6].

The appropriate treatment depends on the location, size, and type of the duodenal cyst, including taking into account patient comorbidities. Complete excision is indicated when the cyst is small and is in no relation with the pancreaticobiliary tree, or when the cyst is complicated with an ulcer, either in the cystic mucosa. Excision is considered the procedure of choice to avoid malignant transformation, as depicted in our patient.

Our case report emphasizes a sudden change in bowel habits, early diagnosis and immediate complete resection of DDS to prevent malignant transformation and extensive surgery. Early treatment may have resulted in a conservative surgical approach, reduced health care cost, and shorter length of hospitalization.

## Conclusions

Duplication cysts in the duodenum are extremely rare, and intestinal duplication cysts are rarely diagnosed in adults. Preoperative diagnosis of primary malignancy in DDC is challenging. A high degree of suspicion is required to diagnose it. However, whenever DDC is suspected, its immediate resection is justified. We report a rare case of duodenal duplication cyst, resulting in malignant transformation which may have been prevented with early resection.
